# Effects of Fertilization and Planting Modes on Soil Organic Carbon and Microbial Community Formation of Tree Seedlings

**DOI:** 10.3390/plants13182665

**Published:** 2024-09-23

**Authors:** Sutong Fan, Yao Tang, Hongzhi Yang, Yuda Hu, Yelin Zeng, Yonghong Wang, Yunlin Zhao, Xiaoyong Chen, Yaohui Wu, Guangjun Wang

**Affiliations:** 1 College of Life and Environmental Sciences, Central South University of Forestry and Technology, Changsha 410004, China; 2 College of Arts and Sciences, Governors State University, University Park, IL 60484, USA; xchen@govst.edu

**Keywords:** tea seed shell biochar, tea meal organic fertilizer, microbial community, soil organic carbon

## Abstract

Biochar and organic fertilizer can significantly increase soil organic carbon (SOC) and promote agricultural production, but it is still unclear how they affect forest SOC after. Here, low-quality plantation soil was subjected to four distinct fertilization treatments: (CK, without fertilization; BC, tea seed shell biochar alone; OF, tea meal organic fertilizer alone; BCF, tea seed shell biochar plus tea meal organic fertilizer). *Cunninghamia lanceolata* (Lamb.) Hook and *Cyclobalanopsis glauca* (Thunb.) Oersted seedlings were then planted in pots at the ratios of 2:0, 1:1, and 0:2 (SS, SQ, QQ) and grown for one year. The results showed that the BCF treatment had the best effect on promoting seedling growth and increasing SOC content. BCF changed soil pH and available nutrient content, resulting in the downregulation of certain oligotrophic groups (*Acidobacteria* and *Basidiomycetes*) and the upregulation of eutrophic groups (*Ascomycota* and *Proteobacteria*). Key bacterial groups, which were identified by Line Discriminant Analysis Effect Size analysis, were closely associated with microbial biomass carbon (MBC) and SOC. Pearson correlation analysis showed that bacterial community composition exhibited a positive correlation with SOC, MBC, available phosphorus, seedling biomass, and plant height, whereas fungal community composition was predominantly positively correlated with seedling underground biomass. It suggested that environmental differences arising from fertilization and planting patterns selectively promote microbial communities that contribute to organic carbon formation. In summary, the combination of biochar and organic fertilizers would enhance the improvement and adaptation of soil microbial communities, playing a crucial role in increasing forest soil organic carbon and promoting tree growth.

## 1. Introduction

As a critical component of global forest resources, plantation forestry has been instrumental in mitigating climate change and enhancing ecological environments [1]. Management measures for plantations usually emphasize improving productivity, biodiversity, and soil health. Common practices include mixed-species planting [2] and fertilization [3]. Continuous research has shown that fertilization measures significantly increased the content of nutrients such as nitrogen, phosphorus, and potassium in the soil, enhancing soil fertility and crop yield sustainability [4]. However, excessive fertilization may lead to soil acidification or alkalization, alter soil pH levels, and consequently impact the ecological functions of microbial communities [5]. Moreover, fertilization measures may also change the structure of the soil, affecting the soil’s water permeability, corrosion resistance, and plant root growth [6]. Using organic fertilizers or soil amendments can balance fertilization to maintain soil fertility and increase organic matter without causing environmental harm. Numerous studies have also demonstrated that integrated fertilizers enhance soil quality and that their prolonged application boosts the relative abundance of beneficial soil microorganisms, thereby improving plant functions [7,8].

As crucial regulators of ecosystem balance and nutrient cycling, soil microorganisms play a vital role in nutrient transformation and significantly contribute to soil fertility and health. This includes key groups such as nitrifying bacteria, denitrifying Pseudomonas, and nitrite carbon-fixing bacteria [9,10,11]. Studies have shown that there is a close relationship between soil microorganisms and aboveground plants. On the one hand, plants generate carbon sources that benefit specific soil microbes, fostering intricate microbial-plant interactions and feedback mechanisms that regulate growth, stability, and diversity [12]. On the other hand, microbes promote plant growth by supplying essential nutrients through metabolic processes [13]. Consequently, forest vegetation influences microbial metabolism, which, in turn, affects the vegetation itself, establishing a feedback system of interactions between soil and forest plants. Changes in the composition of the soil microbial community are often induced by plants, which subsequently influence the growth of neighboring plants and individual soil-cultivating species, a phenomenon known as plant–soil feedback (PSF) [14]. Plants and soil are interdependent; plants regulate the carbon (C) uptake through their roots, while soil microbial activity benefits from rhizodeposition C fluxes. In turn, soil microbes can have direct or indirect effects on plant growth. For instance, plants stimulate above- and below-ground interactions that influence C allocation, rhizodeposition, and microbial growth [15]. Similar to plant-soil feedback, plants also play a crucial role in maintaining species diversity. The complementarity and selection in plant-plant interactions enhance productivity, suggesting that higher plant biodiversity can lead to greater ecosystem stability, improved forest community productivity, and more effective nutrient cycling and soil carbon stocks [16,17,18,19]. Chen et al. [20] demonstrated in a meta-analysis of paired observations from studies on plant mixtures and monocultures that plant diversity increased soil organic carbon. However, interspecific competition among plants can significantly impact the carbon and nitrogen cycles of ecosystems by influencing the transformation processes of these elements mediated by microorganisms. Previous studies have shown that plants can increase microbial biomass carbon, which in turn promotes the carbon mineralization of soil organic matter through intraspecific competition [21]. Other research suggested that intraspecific competition could also intensify competition between plants and microorganisms, which limited the use of nitrogen by microorganisms, limiting nitrogen availability to microorganisms and thereby reducing microbial biomass carbon and enzyme activities in *Fraxinus mandshurica* Rupr and *Cunninghamia lanceolate* (Lamb.) Hook [22].

At present, limited research exists on the impact of fertilization and various planting modes on soil microorganisms. Conducting pot experiments to investigate the effects and mechanisms of fertilizer application and planting modes on soil microbes in forest soils is essential for elucidating the complex interrelationships between plant diversity and soil properties. This research offers a scientific basis for enhancing our comprehension of soil nutrient cycling within forest ecosystems.

In this study, we selected seedlings of *Cunninghamia lanceolate* and *Cyclobalanopsis glauca* (Thunb.) Oersted, representing coniferous and broad-leaved trees, respectively, and paired them in pairs to simulate pure and mixed planting modes. These seedlings were subjected to four types of fertilization to investigate the effects of these factors on soil chemical attributes, soil organic carbon formation, and microbial community structure. The aim was to understand how microorganisms respond to the different combinations of fertilization treatments and tree species, as well as to elucidate the mechanisms by which these factors influenced soil organic carbon (SOC).

## 2. Materials and Methods

### 2.1. Soil, Biochar, and Organic Fertilizer Preparation

Original soil samples were collected from a subsoil horizon at a depth of 45–60 cm at the Qingyang Lake Plantation Forest Factory, located in Hunan Province (28°12′–28°10′20″ N, 111°58′–120°5′0′′ E), in September 2021. The soil is classified as yellow-brown soil with a silty texture (16% clay, 67% sand, and 17% silty sand) and was chosen for its nutrient-poor characteristics. The initial properties of soil are detailed in Table S1. After air-drying, the soil was sieved through a 0.8 cm mesh.

The organic fertilizer was obtained from tea meal powder purchased from Fuming Bio-Organic Fertilizer Co., Ltd., (Shijiazhuang, China). The powder was mixed with surface soil (0–5 cm, sampled from Qingyang Lake Plantation Forest Factory, Changsha, China) and allowed to decompose for 6 months. The ratio of tea meal powder to soil was 9:1. The moisture content was adjusted to 25–35%, and flipped at regular intervals to produce tea meal organic fertilizer. The biochar utilized in the experiment was obtained from Runsheng Biomass Energy Manufacturing Co., Ltd., (Shaoxing, China). Dried *Camellia oleifera* shells were used as raw materials, and pyrolyzed at 600 °C for 4 h to produce biochar. The biochar was sieved through a 0.25 mm sieve before application to soil. The basic information of biochar and organic fertilizer are shown in Table S1.

### 2.2. Experimental Site and Design

The outdoor pot experiment was carried out on a vacant plot at Hunan Botanical Garden (28°12′ N, 112°59′ E), characterized by a subtropical monsoon climate. In 2022, the annual precipitation reached 1361.6 mm, with more than 50% of this rainfall concentrated between May and August. The average annual temperature was recorded at 17.2 °C, with approximately 85 days in the summer experiencing temperatures surpassing 30 °C. The total annual sunshine duration was 1614.3 h, with 59.12% of this sunshine occurring from July to October (www.ceicdata.com, accessed on 12 September 2024).

The total weight of each barrel was 30 kg. There were four different treatments: (1) BC, only tea seed shell biochar was added, the addition amount was 3% (*w*/*w*) [23]; (2) OF, only tea seed meal organic fertilizer was added, the addition amount was 5% (*w*/*w*) [24]; (3) BCF: 3% (*w*/*w*) tea shell biochar + 5% (*w*/*w*) tea seed meal organic fertilizer; (4) CK, the treatment without tea seed shell biochar and tea seed meal organic fertilizer. Each treatment had 6 replicates and incubated for one month to allow nutrients to be released, with regular irrigation to keep soil moisture stable. At the end of October 2021, two seedlings were planted 10 cm apart in each plastic pot (outer diameter: 56 cm, height: 33 cm). Seedlings of *Cyclobalanopsis gilva* and *Cunninghamia lanceolata* of almost equal size and canopy height were planted in three patterns to study the response of mixed and pure planting to soil fertilization. (Young seedlings were selected because the effects of aboveground organic matter on soil physical and chemical characteristics could be excluded as much as possible during the experiment.) Before planting, all roots were thoroughly washed with sterile water. The three planting modes were as follows: (1) SS, with two seedlings of *Cunninghamia lanceolata* planted in a plastic pot; (2) SQ, with one seedling of *Cyclobalanopsis gilva* and one seedling of *Cunninghamia lanceolata* planted in a plastic pot; (3) QQ, with two seedlings of *Cyclobalanopsis gilva* planted in a plastic pot. Therefore, this study has 72 potted plants, specifically three planting groups and four fertilization groups, each treatment has 6 replicates.

### 2.3. Sampling and Determination of Soil Chemical Attributes

Following one year of cultivation, soil samples were collected from the midpoint of the two seedlings of each treatment at a depth of 5–15 cm. These samples were then randomly combined to produce three composite samples per treatment. After removing fine roots, stones, and other impurities, the soil was swiftly sifted through a 2 mm sieve. It was then stored at 4 °C and transported to the laboratory. One portion was air-dried and used for soil chemical attributes measurement, another portion was stored at −20 °C for DNA extraction, and the remaining portion was stored at 4 °C. The average height of each seedling was measured with a ruler. Seedlings were then randomly selected from 3 pots, and their stems were separated from their roots and dried to obtain the biomass. 

Soil pH (1:2.5 soil/water suspensions) was measured using a Sartorius PB-10 (Shanghai Lechen LC-MP-41T) pH meter. SOC was determined by the K_2_Cr_2_O_7_–H_2_SO_4_ digestion method [25,26]. The dissolved organic carbon (DOC) was determined by extracting the soils with deionized water (1:5 ratio) and measuring the C concentration using a TOC analyzer (TOC–V WP, Shimadzu, Japan) [27]. Soil microbial biomass carbon (MBC) was determined using the chloroform fumigation extraction method. In brief, the fumigated and unfumigated soil samples were extracted with K_2_SO_4_, then the extractant was filtered through 0.45 um filter paper, and the dissolved DOC in the filtrate was analyzed by a TOC analyzer [28]. Available phosphorus (AP) was extracted using NH_4_F–HCl and determined by molybdenum blue spectrophotometry and available potassium (AK) was extracted using 1 M NH_4_OAc and determined by fame emission spectrophotometry (FP6430, Shanghai, China) [29]. NH_4_^+^-N and NO_3_^−^-N concentrations were measured using a continuous-flow analyzer (Skalar Analytical, Breda, The Netherlands) [30].

### 2.4. DNA Extraction and Illumina Sequencing

The total DNA of bacteria in the soil was extracted using the TguideS96 Magnetic Genomic DNA kit (Tiangen Biotech, Beijing, China). Primers 338F and 806R were used to amplify the highly denatured V3–V4 region of the bacterial 16S rRNA gene, while primers ITS1F and ITS2R were used to amplify part of the fungal ITS region [31]. All PCR products were quantified and pooled using Quant-iT™ dsDNA HS reagents (Thermo Fisher Scientific, Shanghai, China). High-throughput sequencing procedures were performed by Biomarker Technologies (Biomarker Technologies, Beijing, China) using the Illumina novaseq 6000 (Illumina, San Diego, CA, USA) sequencing strategy on the Illumina Hiseq platform. Reads were denoised into exact sequence variants (OTUs) at = 100% sequence similarity using the DADA2 as implemented in QIIME2 (version 2020.6) [32]. QIIME2 was used to dilute the microbial alpha diversity in each sample, with the number of species observed and Shannon’s index used as a measure of bacterial and fungal diversity [33].

### 2.5. Data Analysis

The data analysis was mainly divided into 5 parts and primarily performed using SPSS version 26.0 for Windows (IBM Corp., Armonk, NY, USA, 2019). First, the differences in plant growth characteristics, soil chemical attributes, and microbial diversity among different planting modes and fertilizer types were analyzed by one-way ANOVA, where *p* < 0.05 indicated significant differences, and the interaction effects of planting patterns and fertilizer types were analyzed by two-way ANOVA, *p* < 0.05 indicated interactive effects. Second, principal coordinate analysis (PCoA) was applied to provide visualization of broad patterns of microbial communities in different treatment groups based on Bray–Curtis distances. Third, Line Discriminant Analysis Effect Size (LEfSe) was used to determine the differentially abundant taxa, and taxa with absolute LDA scores exceeding 3 and *p*-values less than 0.05 were presented. Fourth, the correlations between dominant soil bacterial genera and soil parameters were visualized using Cytoscape. Fifth, the Pearson correlation coefficients > 0.7 and *p* values < 0.01 indicated statistically significant correlations. Mantel tests were employed to assess the associations between microbial community composition and soil variables, as well as seedling growth, using the “vegan” package.

## 3. Results

### 3.1. Effects of Fertilization and Planting Mode on Plants’ Growth

**Figure 1:**
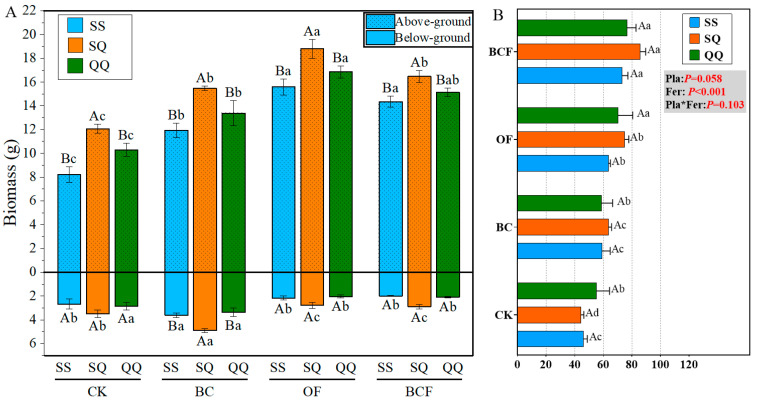
The above and below-ground biomass (**A**) and plant height (**B**) in potted plants by three plant modes (SS, QQ, SQ) treated with four fertilizations (CK, BC, OF, BCF). Data present means (*n* = 3) and standard errors. Each bar represents the average of the growth characteristics of two plants planted in the soil with the corresponding fertilization type, for example, the first blue bar represents the average shoot biomass of two *Cunninghamia lanceolata* seedlings planted in unfertilized soil (CK). Different uppercase and lowercase letters indicate significant differences between different planting patterns and fertilization treatments at *p* < 0.05 level. (SS, QQ, and SQ represent the planting treatment groups: *Cunninghamia lanceolata* + *Cunninghamia lanceolata*, *Cyclobalanopsis gilva* + *Cyclobalanopsis gilva*, *Cyclobalanopsis gilva* + *Cunninghamia lanceolata*, respectively. CK, BC, OF, and BCF represent the fertilization treatment groups: no fertilizer, biochar alone, organic fertilizer alone, and biochar + organic fertilizer, respectively).

The results showed that all three fertilization treatments significantly improved (*p* < 0.05) the shoot biomass of saplings compared to the control group (CK). Interestingly, the single application of biochar increased (*p* < 0.05) the below-ground biomass of seedlings, while the combination of biochar and organic fertilizer (BCF) could regulate the plant height growth of seedlings. Meanwhile, all the above results showed that the significant effect of fertilization treatment on plant growth is particularly obvious in the mixed planting groups (SQ). Additionally, two-way ANOVA analyses showed that fertilization and mixed planting modes had significant (*p* < 0.001) interaction on seedling rhizome growth and no significant interaction on plant height (Figure 1, Table S2).

### 3.2. Effects of Fertilization and Planting Mode on Soil Chemical Attributes

Both mixed planting and fertilizer application could significantly affect soil chemical attributes, and there was a significant (*p* < 0.001) interaction between planting mode and fertilization on soil chemical attributes except AP (two-way ANOVA, *p* = 0.159, Table S3). The influence of fertilization type on improving soil pH was BC > OF > BCF > CK, which could be observed in all planting types. Especially exciting is that the soil pH of mixed planting was higher than that of pure planting in all fertilizer treatments (Table 1). Compared with pure planting, mixed planting decreased soil available nutrients such as AK and AP under all fertilization treatments. Compared with CK, all fertilizer treatments significantly affected the content of available nitrogen and had the highest nitrate and ammonia nitrogen values in OF treatment (except SS), but BC treatment significantly reduced the content of ammonia nitrogen in all planting types. OF and BCF treatments significantly increased the contents of SOC, DOC, and MBC compared with CK and BC treatments, mixed planting was more conducive to the accumulation of SOC and MBC than pure planting, while the DOC contents in mixed planting were lower than that in pure planting.

### 3.3. Effects of Fertilization and Planting Mode on Microbial Community Composition

**Figure 2:**
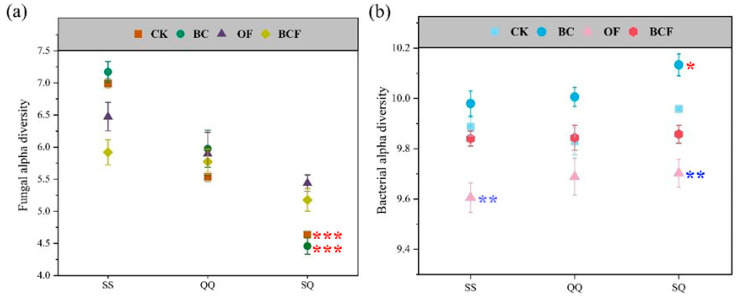
Alpha diversity of microbial community (mean ± SEM). Fungal (**a**) and bacterial α diversity (**b**) in the soil of seedling pots under each treatment (SS, QQ, and SQ represent the planting treatment groups: *Cunninghamia lanceolata* + *Cunninghamia lanceolata*, *Cyclobalanopsis gilva* + *Cyclobalanopsis gilva*, *Cyclobalanopsis gilva* + *Cunninghamia lanceolata*, respectively. CK, BC, OF, and BCF represent the fertilization treatment groups: no fertilizer, biochar alone, organic fertilizer alone, and biochar + organic fertilizer, respectively). The red asterisk and blue asterisk indicate significant diversity among different planting patterns and fertilization treatments, respectively. Note: asterisks denote the significance level. * 0.01 < *p* < 0.05, ** 0.001 < *p* < 0.01, *** *p* < 0.001.

Overall, fungal diversity was not significantly influenced by fertilization treatments. However, mixed planting groups exhibited significantly lower diversity (*p* < 0.001) compared to pure planting groups in CK and BC treatments (Figure 2a). Specifically, OF treatment reduced bacterial diversity, while BC treatment increased it across all planting patterns, with SQ showing the highest value (Figure 2b). Additionally, two-way ANOVA analyses showed that fertilization and mixed planting modes had significant (*p* < 0.001) interaction on bacterial α diversity, but no significant interaction with fungal α diversity (Table S4).

**Figure 3:**
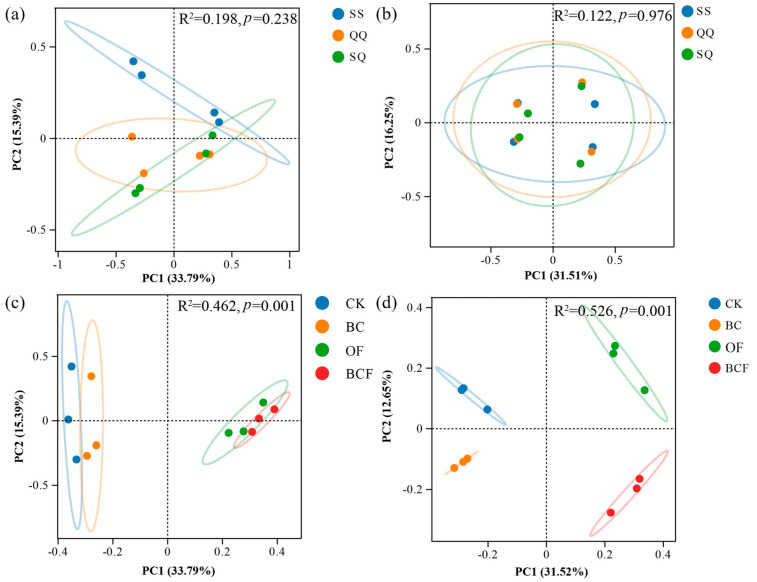
PCoA analysis of fungal (**a**) and bacterial (**b**) community structures in three plantations (SS, QQ, and SQ represent the planting treatment groups: *Cunninghamia lanceolata* + *Cunninghamia lanceolata*, *Cyclobalanopsis gilva* + *Cyclobalanopsis gilva*, *Cyclobalanopsis gilva* + *Cunninghamia lanceolata*, respectively) based on Bray–Curtis distance. PCoA analysis of fungal (**c**) and bacterial (**d**) in four ways of fertilization (CK, BC, OF, and BCF represent the fertilization treatment groups: no fertilizer, biochar alone, organic fertilizer alone, and biochar + organic fertilizer, respectively). The ellipse was drawn assuming a multivariate normal distribution (confidence level: 0.95).

PCoA analysis based on Bray–Curtis distance showed that the fungal and bacterial community structure in potting soil was more affected by fertilization type than planting mode. The bacterial community structure changed more than the fungal community structure between the different fertilization treatments (Figure 3).

**Figure 4:**
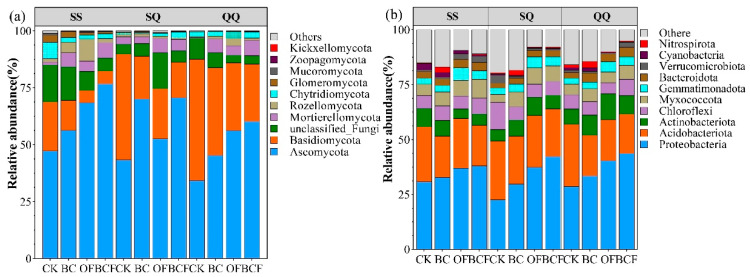
Relative abundance of dominant fungi (**a**) and bacteria (**b**). The figure shows the top 10 phyla in relative abundance, with the rest classified as others (SS, QQ, and SQ represent the planting treatment groups: *Cunninghamia lanceolata* + *Cunninghamia lanceolata*, *Cyclobalanopsis gilva* + *Cyclobalanopsis gilva*, *Cyclobalanopsis gilva* + *Cunninghamia lanceolata*, respectively. CK, BC, OF, and BCF represent the fertilization treatment groups: no fertilizer, biochar alone, organic fertilizer alone, and biochar + organic fertilizer, respectively).

The influences of fertilization and planting modes on the comparative abundances of fungal and bacterial phyla are shown in Figure 4. *Ascomycota* and *Basidiomycota* were the two richest phyla, with total abundance exceeding 68.80%, and the highest reaching 89.84%. However, the relative abundance changes of the two phyla are completely different after fertilization. All three fertilizers (BC, OF, and BCF) could significantly increase (*p* < 0.05) the relative abundance of *Ascomycota* compared to CK, whereas *Basidiomycota* showed the opposite trend. Moreover, the planting mode also has a significant impact on the dominant fungal phyla. Specifically, the relative abundance of *Ascomycetes* in SQ increased significantly (*p* < 0.05) in the BC treatment, while it decreased significantly in the OF treatment (Figure 4a).

The dominant bacterial phyla in all treatments were *Proteobacteria*, *Acidobacteria*, *Actinobacteriota*, and *Chloroflexi*. The richness of *Proteobacteria* and *Actinobacteriota* in the fertilization treatment was significantly higher than that in the CK, whereas the richness of *Acidobacteria* was significantly lower than in that CK treatment. It is to be noted that the richness of *Acidobacteria* in the mixed planting group (SQ) was significantly higher than those in the pure planting group (Figure 4b).

**Figure 5:**
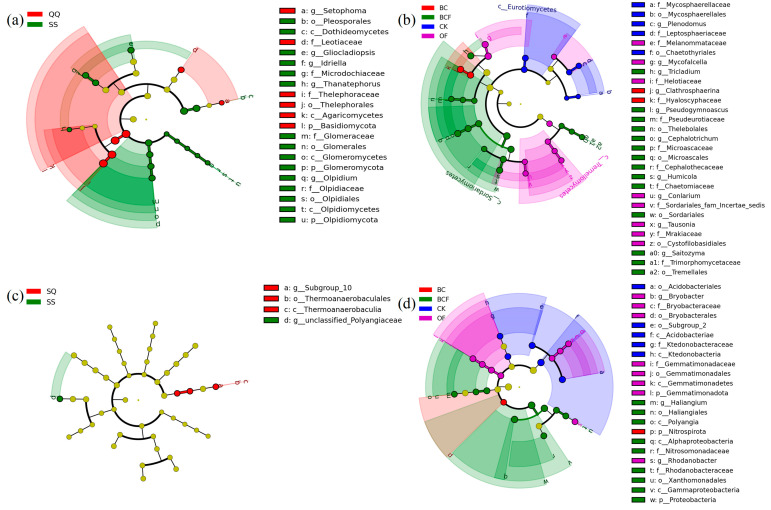
Fungal (**a**,**b**) and bacterial (**c**,**d**) taxa with different changes in plant pots between fertilization, irrespective of plant species (**b**,**d**) and between plant species, irrespective of fertilizer treatment (**c**,**d**) as detected by LEfSe analysis. Taxa with absolute LDA scores over 3 and *p*-values less than 0.05 are shown. The circles radiating from inside to outside represent taxonomic levels from phylum to genus; each small circle at a different taxonomic level represents a taxon at that level, and the diameter of the small circle is proportional to the relative abundance.

LEfSe can be used to color different species according to the group in which the species is most abundant, thereby finding statistically different biomarkers between different groups. Figure 5 further demonstrates that microbial communities are more significantly influenced by fertilization treatments compared to planting modes, with bacteria exhibiting greater sensitivity to environmental changes than fungi. Fertilization results in 23 biomarkers for fungi and 29 biomarkers for bacteria, of which BCF treatment results in nearly half the number of biomarkers. Planting modes result in 21 biomarkers for fungi and four biomarkers for bacteria.

### 3.4. Relationships between Microbial Community Structure and Soil Variables

**Figure 6:**
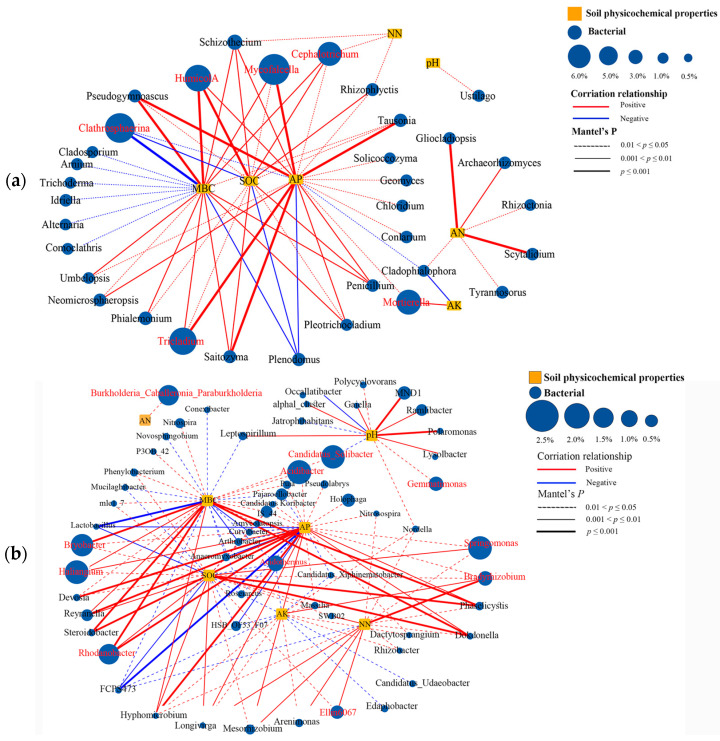
Correlation network analysis showed that soil fungal genera (**a**) and bacterial genera (**b**) that have significant correlations with soil biochemical properties. The red and blue lines indicate positive and negative correlations, respectively.

Network correlation plot provides a visual quantification of fungal genera and bacterial genera that may interact with soil factors (Figure 6). The analysis results demonstrated that *Pseudogymnoascus*, *Humicola*, *Schizothecium*, *Mycofalcella*, *Cephalotrichum*, *Tausonia*, and *Tricladium* were significantly (*p* < 0.05) associated with MBC, SOC, and AP. *Gliocladiopsis*, *Archaeorhizomyces*, and *Scytalidium* were significantly associated with AN. (Figure 6a). *MND1*, *Polaromonas*, *Leptospirillum*, *alphal-cluster*, *Gaiella*, *Ramlibacter*, and *Lysobacter* were significantly (*p* < 0.01) related to soil pH, *Bradyrhizobium*, *Ellin6067*, *Arenimonas*, *Hyphomicrobium* were positively correlated with NN. *Bryobacter*, *Halingium*, *Rhodanobacter*, *Haliangium*, *Phaselicystis*, and *Dokdonella* were significantly associated with MBC, SOC, AP, and NN (Figure 6a). Noticeably, *Clathrosphaerina*, the dominant fungal genus in BC treatment, was negatively correlated with SOC and MBC.

**Figure 7:**
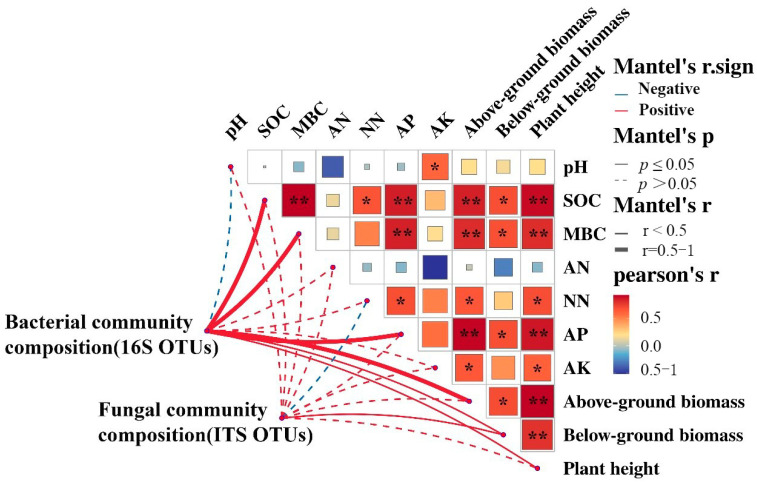
Pairwise comparisons of soil variables shown with a color gradient representing Pearson’s correlation coefficients. “*” and “**” denote the significance level at 0.01 < *p* < 0.05, and *p* < 0.01, respectively. Mantel tests depict the association between taxonomic composition (16 S OTUs, ITS OTUs) and soil variables, as well as seedling growth characteristics. The width of each edge matches Mantel’s r statistic for the equivalent distance correlations. Solid lines indicate significant correlation, and dashed lines indicate insignificant correlation.

Mantel tests analysis showed that the bacterial community composition was positively correlated with SOC, MBC, and AP (r > 0.05, *p* <0.05), and it was beneficial to the growth of three species of seedlings. Fungal community composition had no significant correlation with soil biochemical properties, but it promoted the growth of below-ground biomass of plants, which confirmed that bacterial communities can respond quickly to environmental changes and help plant growth. MBC was closely related to SOC accumulation, and both of them were significantly positively correlated with seedling growth. In addition, seedling growth showed positive associations with available nutrients NN, AP, and AK (Figure 7).

## 4. Discussion

### 4.1. Fertilization and Mixed Planting Mode Impacts on Soil Chemistry

Our results demonstrated that all three fertilization treatments significantly elevate soil pH, with biochar application exerting the most pronounced effect. Furthermore, the impact of mixed planting systems was notably more pronounced than that of pure planting systems. From the perspective of fertilization impacts, the addition of biochar likely enhances soil aggregate formation and stability, improves soil permeability and water retention, and thereby influences soil pH adjustment capabilities [34]. This is corroborated by the observation that biochar treatments consistently yield significantly higher biomass across all planting systems (Figure 1A). Additionally, biochar application may change the structure and activity of soil microbial communities. The metabolism of these microorganisms also influences soil pH, particularly through processes related to the nitrogen cycle. Figure 6b shows the genera that were strongly associated with soil pH. *MND1* represents a key nitrogen-transforming group in carbonaceous soils. Its relative abundance fluctuates with carbon content gradients and correlates with nitrification potential and N_2_O emission [35]. From the perspective of mixed planting systems, root exudates from various plant species can modify soil chemistry, thereby influencing pH levels [36].

The enhancement of soil nutrient availability due to fertilization can reduce the decomposition of soil organic carbon and facilitate its accumulation [37,38]. The research findings indicate that the fertilization treatment enhanced the concentrations of available phosphorus and available potassium. However, the levels of these nutrients were significantly lower (*p* < 0.05) in the mixed sowing treatment compared to the planting treatment (Table 1). Soil organic matter serves as a crucial source of soil available nutrients. Microorganisms decompose it into nutrients accessible to plants through metabolic processes. This decomposition not only enhances microbial biomass but also influences rhizosphere microecology [21]. The positive correlation between MBC, SOC, and AP illustrated in Figure 7 supports this observation. Other studies have shown that organic fertilizer treatment could reduce hydrological loss of N and P loss and improve soil fertility [39,40]. Additionally, rhizosphere effects can explain why the content of available nutrients was lower in mixed plantings [41]. Different plant species may produce different chemical and biological effects in the rhizosphere, such as root exudates, the activity of rhizosphere microorganisms, etc., which can affect the dynamic changes of available nutrients especially soil organic carbon.

Organic fertilizers combined with biochar had previously been shown to improve soil properties [42]. The increase in soil SOC, MBC, and DOC was due to the direct supply of organic matter and nutrients (N, P, and K) in the form of soluble fractions from biochar [43]. In addition, previous studies have shown that organic fertilizer could also improve the ecological environment of the soil [44]. In this study, organic fertilizer combined with biochar additions was more effective in increasing soil SOC, MBC, DOC than organic fertilizer application alone. Differences in biochar particle size also made a difference in improving soil quality, for example, organic carbon and organic matter increased significantly with the addition of biochar combined with 60 mesh giving the best results. It should be noted that the particle size of biochar did not significantly affect the pH [45,46]. The biochar used in this study had a particle size larger than 60 mesh and a lower organic carbon content compared to the organic fertilizer. This likely explains why organic fertilizer is more effective than the biochar combination in increasing soil organic carbon. However, the combined application of biochar and organic fertilizer is not merely additive. SOC, DOC, and MBC content are crucial for managing soil fertility and quality, and they play a potentially significant role in nutrient cycling [47]. The contents were highest in the BCF treatment among all planting methods (Table 1), indicating that the combined use of biochar and organic fertilizer more effectively promotes organic carbon accumulation.

### 4.2. Fertilization and Planting Mode Regulate the Microbial Community Structure

Changes in bacterial and fungal communities reflect corresponding alterations in functional consequences [48]. Specifically, the application of biochar and organic fertilizers has been shown to modify microbial communities in various terrestrial ecosystems, leading to improved soil quality [49,50]. The findings indicated a decrease in the abundance of *Acidobacteria* following the application of organic fertilizer alone and biochar combined with organic fertilizer. This reduction in *Acidobacteria* is associated with an overall improvement in soil quality, which in turn enhances the abundance of copiotrophic bacterial taxa like *Proteobacteria* [51]. *Proteobacteria* showed the highest enrichment and nutrient status in the combined OF and BCF treatments, reflecting higher levels of light and dissolved particulate organic carbon [52]. In contrast, *Acidobacteria* which are known for their role in biogeochemical carbon cycling, were less effective in soils amended with biochar and organic fertilizer. This is attributed to the acidic microenvironment created by these amendments, which is not conducive to *Acidobacteria* metabolism [53]. Similar results were reported by Ren et al. [54], who found that *Acidobacteria* reacted negatively to organic fertilization.

For fungal communities, *Basidiomycota* and *Ascomycota* were thought to react inversely to soil quality [55], as they were broadly classified as oligotrophic and eutrophic [56]. Several studies have explained that in environments with high nutrient levels in the soil, the abundance of *Ascomycota* increased, while that of *Basidiomycota* decreased [57,58]. Previous studies have also shown that soil nutrients may affect fungal communities by mediating surface transformations in plants and that soil fungi prefer neutral to acidic environments [59,60]. Similarly, we found that the addition of biochar and organic fertilizer favored *Ascomycota* over *Basidiomycota*. LEfse results indicated *Ascomycota* and *Basidiomycota* varied considerably after organic fertilization. However, there were exceptions to this trend. Several fungi within *Basidiomycota*, such as *Micropsalliota*, *Tausonia*, and *Saitozyma* showed a positive response to the fertilizer treatment.

Fungi participate in the carbon cycle mainly by secreting degrading enzymes, absorption, and metabolism [61]. Cellulase, xylanase, etc. produced by fungi decompose complex organic substances such as cellulose and hemicellulose in plant cell walls, convert them into small molecular organic substances, and release carbon. *Pseudogymnoascus Mycofalcella, Schizothecium, Cephalotrichum*, and *Tricladium* are a group of fungi found to be highly productive in enzymes related to cellulose, xylan, and wax degradation [62,63,64,65]. Their abundance was found to be significantly positively correlated with SOC and MBC accumulation (Figure 6a). In addition, some bacteria such as *Bryobacter*, *Phaselicystis*, and *Dokdonella* were some nitrogen-fixing that closely related to SOC (Figure 6b). The activities of nitrogen-fixing bacteria increase the nitrogen content in the soil, allowing plants to grow better and absorb more carbon dioxide. This highlighted the significant role of nitrogen-fixing bacteria in maintaining the ecological balance of carbon and nitrogen within ecosystems [66].

### 4.3. Close Links between Soil Organic Carbon, Microorganisms, and Below-Ground Biomass

The accumulation of SOC is hypothesized to result from increased belowground inputs, such as root litter and/or root exudates, or from reduced microbial-mediated losses of organic carbon [67,68]. The latter scenario suggests a heightened efficiency in soil microbial carbon turnover. In a similar vein, our results demonstrate that the application of biochar, organic fertilizer, and a combination of both significantly enhances root biomass and soil microbial biomass carbon in seedlings (Figure 1 and Table 1). Increased plant carbon input and accelerated microbial growth may be mechanistically linked to the enhanced accumulation of SOC. Consistent with our expectations, the strong correlation observed between underground biomass, MBC, and SOC in the correlation network analysis (Figure 7) further supported this hypothesis. Yang et al. [69] reported that under increased nitrogen deposition, the root pathway promoted SOC accumulation, but the microbial metabolism reduced the accumulation. In contrast, the mycelial pathway positively influenced SOC accumulation, as fungi consuming the same substrate produced more biomass and thus stored more organic carbon. Soil fungi were reported to have twice the biomass of bacteria [70] and exhibit high adaptability to soil conditions [71]. As we mentioned, fertilization could enrich key fungi groups such as *Pseudogymnoascus*, *Mycofalcella*, *Schizothercium*, *Cephalotrichum*, and *Tricladium*. These organisms are capable of thriving by synthesizing enzymes that degrade carbon-rich substrates characterized by lower bioavailability and more complex structures [72]. The slow decomposition of recalcitrant organic matter facilitated the accumulation of more microbial biomass carbon, which was undoubtedly positive for the indirect accumulation of organic carbon. This phenomenon was also observable in slow-growing oligotrophic organisms like *Acidobacteria* (*Bryobacter*), whose metabolic diversity allowed them to degrade complex substrates originating from resistant soil organic matter reservoirs [73,74]. Shi et al. [75] found that applying organic fertilizers increased organic substances like organic acids, sugars, alcohols, and amino acids, which enriched bacterial communities involved in nutrient cycling, plant productivity, and disease suppression, including *Rhodanobacteria, Bryobacter*, and *Sphingomonas*. This finding corroborated our results, demonstrating that these Gram-negative bacteria show significant positive correlations with both SOC and MBC, which underscored that these pivotal bacterial groups were capable of producing increased biomass through the metabolism of readily decomposable substrates, thereby enhancing organic carbon storage.

## 5. Conclusions

This study represented the inaugural use of tea meal organic fertilizer and biochar across three planting systems of *Cunninghamia lanceolata* and *Cyclobalanopsis glauca*, assessing their impact on soil microbial communities and organic carbon. We found that microorganisms were more affected by fertilization than planting modes with bacteria being particularly sensitive. Fertilization-induced variations in bacterial communities primarily enhance organic carbon accumulation by increasing microbial biomass carbon through the metabolism of readily degradable substrates. While fungi tend to enhance the accumulation of SOC by influencing belowground plant biomass. This knowledge was essential for a comprehensive understanding of plant-soil-microbe relationships.

## Figures and Tables

**Table 1:** Soil chemical attributes under different planting modes and different fertilization patterns.

Planting Mode	Fertilization Patterns	pH	SOC	DOC	MBC	NH_4_^+^-N	NO_3_^−^-N	AP	AK
			(g·kg^−1^)	(g·kg^−1^)	(mg·kg^−1^)	(mg·kg^−1^)	(mg·kg^−1^)	(g·kg^−1^)	(g·kg^−1^)
SS	CK	4.87 ^Ca^	9.31 ^Ca^	1.59 ^Ca^	223.10 ^Cb^	8.39 ^Aa^	1.3 ^Db^	0.25 ^Ca^	1.16 ^Db^
	BC	5.89 ^Ab^	10.64 ^Cb^	1.69 ^Ca^	208.87 ^Ca^	1.71 ^Cc^	4.32 ^Bb^	0.39 ^BCa^	2.76 ^Aa^
	OF	4.98 ^Cb^	16.96 ^Bb^	2.27 ^Bb^	339.85 ^Bb^	3.2 ^Bb^	3.65 ^Cc^	0.59 ^ABb^	1.99 ^Cb^
	BCF	5.31 ^Ba^	23.28 ^Ac^	2.91 ^Aa^	410.82 ^Ab^	3.01 ^Bc^	20.93 ^Ab^	0.78 ^Aa^	2.47 ^Bb^
SQ	CK	4.88 ^Da^	11.97 ^Ca^	1.57 ^Ba^	281.05 ^Ca^	2.65 ^Cc^	1.19 ^Cb^	0.26 ^Da^	0.97 ^Dc^
	BC	6.00 ^Aa^	12.64 ^Ca^	1.61 ^Ba^	236.68 ^Da^	2.05 ^Db^	4.81 ^Bb^	0.36 ^Ca^	2.49 ^Ab^
	OF	5.03 ^Ca^	23.28 ^Ba^	2.18 ^Ab^	366.47 ^Ba^	4.27 ^Ba^	5.1 ^Bb^	0.55 ^Bc^	1.91 ^Cc^
	BCF	5.41 ^Ba^	39.24 ^Aa^	2.17 ^Ab^	442.40 ^Aa^	4.89 ^Aa^	31.43 ^Aa^	0.74 ^Aa^	2.39 ^Bc^
QQ	CK	4.66 ^Cb^	8.65 ^Ca^	1.68 ^Ca^	198.63 ^Cb^	3.03 ^Cb^	16.97 ^Ba^	0.33 ^Ca^	1.65 ^Da^
	BC	5.38 ^Ac^	9.31 ^Cb^	1.63 ^Ca^	206.50 ^Ca^	2.36 ^Ba^	18.39 ^Aa^	0.42 ^Ba^	2.82 ^Aa^
	OF	4.63 ^Cc^	24.61 ^Ba^	2.43 ^Ba^	329.26 ^Bb^	3.15 ^Bb^	18.59 ^Aa^	0.78 ^Aa^	2.47 ^Ca^
	BCF	5.22 ^Ba^	29.26 ^Ab^	2.79 ^Aa^	391.96 ^Ab^	3.37 ^Ab^	19.55 ^Ab^	0.79 ^Aa^	2.65 ^Ba^

Note: SOC (g/kg): soil organic carbon; DOC (g/kg): dissolved organic carbon; MBC (mg/kg): soil microbial biomass; NH_4_^+^-N (mg/kg): ammonium nitrogen; NO_3_^−^-N (mg/kg): nitrate nitrogen; AP (g/kg): available phosphorus, AK (g/kg): available potassium; the values in each horizontal line represent the chemical properties of the soil with different fertilization types where the corresponding plants are planted. For example, the first value represents the pH value in the unfertilized soil (CK) where two Chinese fir trees are planted. Values of soil chemical attributes are means, *n* = 3, and the capital and lowercase letters followed in the same row were statistically significant in different fertilization patterns and planting modes at *p* < 0.05, respectively. (SS, QQ, and SQ represent the planting treatment groups: *Cunninghamia lanceolata* + *Cunninghamia lanceolata*, *Cyclobalanopsis gilva* + *Cyclobalanopsis gilva*, *Cyclobalanopsis gilva* + *Cunninghamia lanceolata*, respectively. CK, BC, OF, and BCF represent the fertilization treatment groups: no fertilizer, biochar alone, organic fertilizer alone, and biochar + organic fertilizer, respectively).

## Data Availability

All data in this study are included in the article and Supplementary Materials; further inquiries can be directed to the corresponding author.

## Supplementary Materials

The following supporting information can be downloaded at: https://www.mdpi.com/article/10.3390/plants13182665/s1, Table S1: Basic properties of soil, biochar, and organic fertilizer. Table S2: Effects of plant-plant interactions and fertilization on seedling growth (two-way ANOVA). Table S3: Effects of plant-plant interactions and fertilization on soil properties (two-way ANOVA). Table S4: Effects of plant-plant interactions and fertilization on microbial diversity (two-way ANOVA).

## References

[1] Busch J., Bukoski J.J., Cook-Patton S.C., Griscom B., Kaczan D., Potts M.D., Yi Y., Vincent J.R. (2024). Cost-effectiveness of natural forest regeneration and plantations for climate mitigation. Nat. Clim. Chang..

[2] Liu Y., Lei P., Xiang W., Yan W., Chen X. (2017). Accumulation of soil organic C and N in planted forests fostered by tree species mixture. Biogeosciences.

[3] Ren T., Liao J., Delgado–Baquerizo M., Ni J., Li Y., Jin L., Ruan H. (2023). Organic fertilization promotes the accumulation of soil particulate organic carbon in a 9–year plantation experiment. Land. Degrad. Dev..

[4] Qaswar M., Huang J., Ahmed W., Li D., Liu S., Zhang L., Cai A., Liu L., Xu Y., Gao J. (2020). Yield sustainability, soil organic carbon sequestration and nutrients balance under long-term combined application of manure and inorganic fertilizers in acidic paddy soil. Soil Tillage Res..

[5] Wang J.L., Liu K.L., Zhao X.Q., Zhang H.Q., Li D., Li J.J., Shen R.F. (2021). Balanced fertilization over four decades has sustained soil microbial communities and improved soil fertility and rice productivity in red paddy soil. Sci. Total Environ..

[6] Zhang Y.J., Ye C., Su Y.W., Peng W.C., Lu R., Liu Y.X., Huang H.C., He X.H., Yang M., Zhu S.S. (2022). Soil Acidification caused by excessive application of nitrogen fertilizer aggravates soil-borne diseases: Evidence from literature review and field trials. Agric. Ecosyst. Environ..

[7] Pereg L., Morugán-Coronado A., McMillan M., García-Orenes F. (2018). Restoration of nitrogen cycling community in grapevine soil by a decade of organic fertilization. Soil Tillage Res..

[8] Liu J., Shu A., Song W., Shi W., Li M., Zhang W., Li Z., Liu G., Yuan F., Zhang S. (2021). Long-term organic fertilizer substitution increases rice yield by improving soil properties and regulating soil bacteria. Geoderma.

[9] Pachiadaki M.G., Sintes E., Bergauer K., Brown J.M., Record N.R., Swan B.K., Mathyer M.E., Hallam S.J., Lopez-Garcia P., Takaki Y. (2017). Major role of nitrite-oxidizing bacteria in dark ocean carbon fixation. Science.

[10] Koch R.A., Yoon G.M., Aryal U.K., Lail K., Amirebrahimi M., LaButti K., Lipzen A., Riley R., Barry K., Henrissat B. (2021). Symbiotic nitrogen fixation in the reproductive structures of a basidiomycete fungus. Curr. Biol..

[11] Tang S., Ma Q., Luo J., Xie Y., Hashmi M.L.u.R., Pan W., Zheng N., Liu M., Wu L. (2021). The inhibition effect of tea polyphenols on soil nitrification is greater than denitrification in tea garden soil. Sci. Total Environ..

[12] Eldridge D.J., Travers S.K., Val J., Ding J., Wang J.T., Singh B.K., Delgado–Baquerizo M., Chapman S. (2021). Experimental evidence of strong relationships between soil microbial communities and plant germination. J. Ecol..

[13] Das P.P., Singh K.R.B., Nagpure G., Mansoori A., Singh R.P., Ghazi I.A., Kumar A., Singh J. (2022). Plant-soil-microbes: A tripartite interaction for nutrient acquisition and better plant growth for sustainable agricultural practices. Environ. Res..

[14] Bever J.D. (2003). Soil community feedback and the coexistence of competitors: Conceptual frameworks and empirical tests. New Phytol..

[15] Zhang K., Maltais-Landry G., Liao H.-L. (2021). How soil biota regulate C cycling and soil C pools in diversified crop rotations. Soil Biol. Biochem..

[16] Haghverdi K., Kooch Y. (2019). Effects of diversity of tree species on nutrient cycling and soil–related processes. Catena.

[17] Jing J., Bezemer T.M., van der Putten W.H., Power A. (2015). Complementarity and selection effects in early and mid-successional plant communities are differentially affected by plant–soil feedback. J. Ecol..

[18] Wagg C., O’Brien M.J., Vogel A., Scherer–Lorenzen M., Eisenhauer N., Schmid B., Weigelt A. (2017). Plant diversity maintains long-term ecosystem productivity under frequent drought by increasing short–term variation. Ecology.

[19] Zheng H., Guber A.K., Kuzyakov Y., Zhang W., Kravchenko A.N. (2022). Plant species and plant neighbor identity affect associations between plant assimilated C inputs and soil pores. Geoderma.

[20] Chen C., Chen H.Y.H., Chen X., Huang Z. (2019). Meta-analysis shows positive effects of plant diversity on microbial biomass and respiration. Nat. Commun..

[21] Sun Y., Zang H., Splettstößer T., Kumar A., Xu X., Kuzyakov Y., Pausch J. (2020). Plant intraspecific competition and growth stage alter carbon and nitrogen mineralization in the rhizosphere. Plant Cell Environ..

[22] Yin L., Dijkstra F.A., Wang P., Zhu B., Cheng W. (2018). Rhizosphere priming effects on soil carbon and nitrogen dynamics among tree species with and without intraspecific competition. New Phytol..

[23] Song Q., He Y., Wu Y., Chen S., Zhang T., Chen H. (2020). Biochar Impacts on Acidic Soil from Camellia Oleifera Plantation: A Short-Term Soil Incubation Study. Agronomy.

[24] Zhaoxiang W., Huihu L., Qiaoli L., Changyan Y., Faxin Y. (2020). Application of bio-organic fertilizer, not biochar, in degraded red soil improves soil nutrients and plant growth. Rhizosphere.

[25] Sorrell B.K., Brix H., Schierup H.-H., Lorenzen B. (1997). Die-back of Phragmites australis: Influence on the distribution and rate of sediment methanogenesis. Biogeochemistry.

[26] Bai J., Ouyang H., Deng W., Zhu Y., Zhang X., Wang Q. (2005). Spatial distribution characteristics of organic matter and total nitrogen of marsh soils in river marginal wetlands. Geoderma.

[27] Jones D., Willett V. (2006). Experimental evaluation of methods to quantify dissolved organic nitrogen (DON) and dissolved organic carbon (DOC) in soil. Soil Biol. Biochem..

[28] Brookes P.C., Landman A., Pruden G., Jenkinson D.S. (1985). Chloroform fumigation and the release of soil nitrogen: A rapid direct extraction method to measure microbial biomass nitrogen in soil. Soil Biol. Biochem..

[29] Bray R.H., Kurtz L.T. (1945). Determination of Total, Organic, and Available Forms of Phosphorus in Soils. Soil Sci..

[30] Brooks P.D., Stark J.M., McInteer B.B., Preston T. (1989). Diffusion Method to Prepare Soil Extracts for Automated Nitrogen–15 Analysis. Soil Sci. Soc. Am. J..

[31] Knight R., Vrbanac A., Taylor B.C., Aksenov A., Callewaert C., Debelius J., Gonzalez A., Kosciolek T., McCall L.-I., McDonald D. (2018). Best practices for analysing microbiomes. Nat. Rev. Microbiol..

[32] Callahan B.J., McMurdie P.J., Rosen M.J., Han A.W., Johnson A.J.A., Holmes S.P. (2016). DADA2: High-resolution sample inference from Illumina amplicon data. Nat. Methods.

[33] Caporaso J.G., Kuczynski J., Stombaugh J., Bittinger K., Bushman F.D., Costello E.K., Fierer N., Peña A.G., Goodrich J.K., Gordon J.I. (2010). QIIME allows analysis of high-throughput community sequencing data. Nat. Methods.

[34] Shi R.Y., Li J.Y., Ni N., Xu R.K. (2019). Understanding the biochar’s role in ameliorating soil acidity. J. Integr. Agric..

[35] Yu M.J., Su W.Q., Huang L.B., Parikh S.J., Tang C.X., Dahlgren R.A., Xu J.M. (2021). Bacterial community structure and putative nitrogen-cycling functional traits along a charosphere gradient under waterlogged conditions. Soil Biol. Biochem..

[36] Liu Y., Evans S.E., Friesen M.L., Tiemann L.K. (2022). Root exudates shift how N mineralization and N fixation contribute to the plant-available N supply in low fertility soils. Soil Biol. Biochem..

[37] Jílková V., Sim A., Thornton B., Paterson E. (2023). Grass rather than legume species decreases soil organic matter decomposition with nutrient addition. Soil Biol. Biochem..

[38] Khalsa S.D.S., Hart S.C., Brown P.H. (2022). Nutrient dynamics from surface-applied organic matter amendments on no-till orchard soil. Soil Use Manag..

[39] Bah H.D., Zhou M.H., Ren X., Hu L., Dong Z.X., Zhu B. (2020). Effects of organic amendment applications on nitrogen and phosphorus losses from sloping cropland in the upper Yangtze River. Agric. Ecosyst. Environ..

[40] Zhang G., Zhou G., Zhou X., Zhou L., Shao J., Liu R., Gao J., He Y., Du Z., Tang J. (2023). Effects of tree mycorrhizal type on soil respiration and carbon stock via fine root biomass and litter dynamic in tropical plantations. J. Plant Ecol..

[41] Hinsinger P., Bengough A.G., Vetterlein D., Young I.M. (2009). Rhizosphere: Biophysics, biogeochemistry and ecological relevance. Plant Soil.

[42] Chen L.M., Li X.Y., Peng Y.T., Xiang P., Zhou Y.Z., Yao B., Zhou Y.Y., Sun C.R. (2022). Co-application of biochar and organic fertilizer promotes the yield and quality of red pitaya (*Hylocereus polyrhizus*) by improving soil properties. Chemosphere.

[43] Sun Y.Q., Xiong X.N., He M.J., Xu Z.B., Hou D.Y., Zhang W.H., Ok Y.S., Rinklebe J., Wang L.L., Tsang D.C.W. (2021). Roles of biochar-derived dissolved organic matter in soil amendment and environmental remediation: A critical review. Chem. Eng. J..

[44] Cheng H.Y., Zhang D.Q., Huang B., Song Z.X., Ren L.R., Hao B.Q., Liu J., Zhu J.H., Fang W.S., Yan D.D. (2020). Organic fertilizer improves soil fertility and restores the bacterial community after 1,3-dichloropropene fumigation. Sci. Total Environ..

[45] Zeeshan M., Ahmad W., Hussain F., Ahamd W., Numan M., Shah M., Ahmad I. (2020). Phytostabalization of the heavy metals in the soil with biochar applications, the impact on chlorophyll, carotene, soil fertility and tomato crop yield. J. Clean. Prod..

[46] Gu Y.-y., Zhang H.-y., Liang X.-y., Fu R., Li M., Chen C.-j. (2022). Effect of different biochar particle sizes together with bio-organic fertilizer on rhizosphere soil microecological environment on saline–alkali land. Front. Environ. Sci..

[47] Merante P., Dibari C., Ferrise R., Sánchez B., Iglesias A., Lesschen J.P., Kuikman P., Yeluripati J., Smith P., Bindi M. (2017). Adopting soil organic carbon management practices in soils of varying quality: Implications and perspectives in Europe. Soil Tillage Res..

[48] Ai C., Zhang S.Q., Zhang X., Guo D.D., Zhou W., Huang S.M. (2018). Distinct responses of soil bacterial and fungal communities to changes in fertilization regime and crop rotation. Geoderma.

[49] Su L.X., Bai T.Y., Wu G., Zhao Q.Y., Tan L.H., Xu Y.D. (2022). Characteristics of soil microbiota and organic carbon distribution in jackfruit plantation under different fertilization regimes. Front. Microbiol..

[50] Wang Y.F., Chen P., Wang F.H., Han W.X., Qiao M., Dong W.X., Hu C.S., Zhu D., Chu H.Y., Zhu Y.G. (2022). The ecological clusters of soil organisms drive the ecosystem multifunctionality under long-term fertilization. Environ. Int..

[51] Duan Y.M., Zhang L.S., Yang J.F., Zhang Z.Q., Awasthi M.K., Li H.K. (2022). Insight to bacteria community response of organic management in apple orchard-bagasse fertilizer combined with biochar. Chemosphere.

[52] Duan Y.M., Yang J.F., Song Y.F., Chen F.N., Li X.F., Awasthi M.K., Li H.K., Zhang L.S. (2021). Clean technology for biochar and organic waste recycling, and utilization in apple orchard. Chemosphere.

[53] Kalam S., Basu A., Ahmad I., Sayyed R.Z., El-Enshasy H.A., Dailin D.J., Suriani N.L. (2020). Recent Understanding of Soil Acidobacteria and Their Ecological Significance: A Critical Review. Front. Microbiol..

[54] Ren J.H., Liu X.L., Yang W.P., Yang X.X., Li W.G., Xia Q., Li J.H., Gao Z.Q., Yang Z.P. (2021). Rhizosphere soil properties, microbial community, and enzyme activities: Short-term responses to partial substitution of chemical fertilizer with organic manure. J. Environ. Manag..

[55] Li Z.D., Jiao Y.Q., Yin J., Li D., Wang B.B., Zhang K.L., Zheng X.X., Hong Y., Zhang H.X., Xie C. (2021). Productivity and quality of banana in response to chemical fertilizer reduction with bio-organic fertilizer: Insight into soil properties and microbial ecology. Agric. Ecosyst. Environ..

[56] Ho A., Di Lonardo D.P., Bodelier P.L.E. (2017). Revisiting life strategy concepts in environmental microbial ecology. FEMS Microbiol. Ecol..

[57] Nie S.A., Lei X.M., Zhao L.X., Brookes P.C., Wang F., Chen C.R., Yang W.H., Xing S.H. (2018). Fungal communities and functions response to long-term fertilization in paddy soils. Appl. Soil Ecol..

[58] Semenov M.V., Krasnov G.S., Semenov V.M., van Bruggen A. (2022). Mineral and Organic Fertilizers Distinctly Affect Fungal Communities in the Crop Rhizosphere. J. Fungi.

[59] Chu H.Y., Xiang X.J., Yang J., Adams J.M., Zhang K.P., Li Y.T., Shi Y. (2016). Effects of Slope Aspects on Soil Bacterial and Arbuscular Fungal Communities in a Boreal Forest in China. Pedosphere.

[60] Hu X.J., Liu J.J., Wei D., Zhu P., Cui X., Zhou B.K., Chen X.L., Jin J., Liu X.B., Wang G.H. (2017). Effects of over 30-year of different fertilization regimes on fungal community compositions in the black soils of northeast China. Agric. Ecosyst. Environ..

[61] Boddy L. (2016). Fungi, Ecosystems, and Global Change. The Fungi.

[62] Padhi S., Dias I., Korn V.L., Bennett J.W. (2018). Causative Agent of White-Nose Syndrome in Bats Is Inhibited by Safe Volatile Organic Compounds. J. Fungi.

[63] Ohm R.A., de Jong J.F., Lugones L.G., Aerts A., Kothe E., Stajich J.E., de Vries R.P., Record E., Levasseur A., Baker S.E. (2010). Genome sequence of the model mushroom *Schizophyllum commune*. Nat. Biotechnol..

[64] Miyauchi S., Kiss E., Kuo A., Drula E., Kohler A., Sánchez-García M., Morin E., Andreopoulos B., Barry K.W., Bonito G. (2020). Large-scale genome sequencing of mycorrhizal fungi provides insights into the early evolution of symbiotic traits. Nat. Commun..

[65] Uday U.S.P., Majumdar R., Tiwari O.N., Mishra U., Mondal A., Bandyopadhyay T.K., Bhunia B. (2017). Isolation, screening and characterization of a novel extracellular xylanase from *Aspergillus niger* (*KP874102.1*) and its application in orange peel hydrolysis. Int. J. Biol. Macromol..

[66] Kuypers M.M.M., Marchant H.K., Kartal B. (2018). The microbial nitrogen-cycling network. Nat. Rev. Microbiol..

[67] Liang G.P., Stark J., Waring B.G. (2023). Mineral reactivity determines root effects on soil organic carbon. Nat. Commun..

[68] Prommer J., Walker T.W.N., Wanek W., Braun J., Zezula D., Hu Y., Hofhansl F., Richter A. (2019). Increased microbial growth, biomass, and turnover drive soil organic carbon accumulation at higher plant diversity. Glob. Chang. Biol..

[69] Zhu X., Zhang Z., Wang Q., Peñuelas J., Sardans J., Lambers H., Li N., Liu Q., Yin H., Liu Z. (2022). More soil organic carbon is sequestered through the mycelium pathway than through the root pathway under nitrogen enrichment in an alpine forest. Glob. Chang. Biol..

[70] Bar-On Y.M., Phillips R., Milo R. (2018). The biomass distribution on Earth. Proc. Natl. Acad. Sci. USA.

[71] Fierer N., Strickland M.S., Liptzin D., Bradford M.A., Cleveland C.C. (2009). Global patterns in belowground communities. Ecol. Lett..

[72] Bahram M., Hildebrand F., Forslund S.K., Anderson J.L., Soudzilovskaia N.A., Bodegom P.M., Bengtsson-Palme J., Anslan S., Coelho L.P., Harend H. (2018). Structure and function of the global topsoil microbiome. Nature.

[73] Rasche F., Knapp D., Kaiser C., Koranda M., Kitzler B., Zechmeister-Boltenstern S., Richter A., Sessitsch A. (2011). Seasonality and resource availability control bacterial and archaeal communities in soils of a temperate beech forest. ISME J..

[74] Lladó S., López-Mondéjar R., Baldrian P. (2017). Forest Soil Bacteria: Diversity, Involvement in Ecosystem Processes, and Response to Global Change. Microbiol. Mol. Biol. R..

[75] Shi R., Wang S., Xiong B.J., Gu H.Y., Wang H.L., Ji C., Jia W.J., Horowitz A.R., Zhen W.J., Ben Asher J. (2022). Application of Bioorganic Fertilizer on *Panax notoginseng* Improves Plant Growth by Altering the Rhizosphere Microbiome Structure and Metabolism. Microorganisms.

