# Supplementary material for: Effects of Fertilization and Planting Modes on Soil Organic Carbon and Microbial Community Formation of Tree Seedlings
Source: Plants (Basel). 2024 Sep 23;13(18):2665. doi: 10.3390/plants13182665 (PMC11434958; doi:10.3390/plants13182665)
Supplement: Supplementary file 1 [file plants-13-02665-s001.zip › plants-3171078-supplementary.pdf]

Supplementary Table

Table S1 Basic properties of soil, biochar, and organic fertilizer.

sample	pH	TN (g • kg^{-1})	TP (g • kg^{-1})	TK (g • kg^{-1})	SOC (g • kg^{-1})
Initial soil	4.42	0.48	0.25	17.64	5.19
Tea-seed shells biochar	8.24	5.13	3.75	46.35	332.51
tea meal organic fertilizer	7.2	5.54	4.55	32.65	421.33

Note: TN (g·kg-1): Total nitrogen; TP (g·kg-1): Total phosphorus; TK(g·kg-1): Total potassium; SOC (g·kg-1): soil organic carbon.

Table S2 Effects of plant-plant interactions and fertilization on seedling growth (two-way ANOVA).

		Above-ground biomass	Below-ground biomass	Plant height
Fertilization	F	238.76	16.14	4405.06
	P	<0.001	<0.001	<0.001
Planting method	F	61.90	6.56	286.52
	P	<0.001	<0.001	0.058
Fertilization * Planting method	F	2.92	0.86	417.48
	P	<0.001	<0.001	0.103

Table S3 Effects of plant-plant interactions and fertilization on soil properties (two-way ANOVA).

		pH	SOC	AP	AK	AN	NN	DOC	MBC
Fertilization	F	661.027	490.062	109.9	961.321	903.75	2649.392	195.21	155.82
	P	<0.001	<0.001	<0.001	<0.001	<0.001	<0.001	<0.001	<0.001

Planting method	F	165.539	72.062	8.405	5360.344	303.802	1738.281	32.54	42.03
	P	<0.001	<0.001	0.002	<0.001	<0.001	<0.001	<0.001	<0.001
Fertilization * Planting method	F	14.292	23.062	1.72	68.240	882.51	613.846	21.64	33.26
	P	<0.001	<0.001	0.159	<0.001	<0.001	<0.001	<0.001	<0.001

Note: SOC (g/kg): soil organic carbon; DOC (g/kg): dissolved organic carbon; MBC (mg/kg): soil microbial biomass; NH_4^+-N (mg/kg): ammonium nitrogen; NO_3^--N (mg/kg): nitrate nitrogen; AP (g/kg): available phosphorus, AK (g/kg): available potassium.

Table S4 Effects of plant-plant interactions and fertilization on microbial diversity (two-way ANOVA).

		α diversity of bacteria	α diversity of fungi
Fertilization	F	0.517	0.772
	P	<0.001	0.052
Planting method	F	0.072	17.88
	P	<0.001	0.084
Fertilization * Planting method	F	0.078	5.65
	P	<0.001	<0.001
